# Radar detection of pedestrian-induced vibrations on Michelangelo's David

**DOI:** 10.1371/journal.pone.0174480

**Published:** 2017-04-10

**Authors:** Massimiliano Pieraccini, Michele Betti, Davide Forcellini, Devis Dei, Federico Papi, Gianni Bartoli, Luca Facchini, Riccardo Corazzi, Vladimir Cerisano Kovacevic

**Affiliations:** 1 Department of Information Engineering (DINFO), University of Florence, Florence, Italy; 2 Department of Civil and Environmental Engineering (DICEA), University of Florence, Florence, Italy; 3 University of San Marino, San Marino, Republic of San Marino; 4 Florence Engineering s.r.l., Florence, Italy; Universita degli Studi di Verona, ITALY

## Abstract

This paper summarizes the results of a two-day dynamic monitoring of Michelangelo's David subject to environmental loads (city traffic and pedestrian loading induced by tourists visiting the Accademia Gallery). The monitoring was carried out by a no-contact technique using an interferometric radar, whose effectiveness in measuring the resonant frequencies of structures and historic monuments has proved over the last years through numerous monitoring activities. Owing to the dynamic behavior of the measurement system (radar and tripod), an accelerometer has been installed on the radar head to filter out the movement component of the measuring instrument from the measurement of the David's displacement. Measurements were carried out in the presence and absence of visitors, to assess their influence on the dynamic behavior of the statue. A numerical model of the statue was employed to evaluate the experimental results.

## Introduction

Every year about a million and a half tourists visit the Michelangelo’s David in the Accademia Gallery in Florence (Italy). Such a large number (about four thousand people each opening day) in the last few years caused much concern (or alarms) over the effects of visitors' footfall vibrations on the statue’s safety. The problem is not irrelevant in principle, as the statue is known to have been historically affected by a well-known system of visible cracks on the legs: a first system can be seen in the tree trunk that supports the right leg, and a second one in the lower part of the left leg. The cracks were first observed between 1852 and 1872, when a growing concern arose around the David's deterioration and safety. At that time, the statue was still located in front of the main entrance of Florence town hall (“Palazzo Vecchio”) where, after a long debate, it had been placed on September 8, 1504. Three Committees were established, and the conclusive decision of the last one, chaired by Luigi Federico Menabrea (1809–1896), was to protect Michelangelo’s David by moving it inside the Accademia Gallery ([1–2]).

As a result of Michelangelo’s conception, David stands with one leg holding its full weight (the right one) and the other leg (the left one) forward. Such a static behavior, with the right leg bearing most of the David's weight was clear to Michelangelo who decided to reinforce the right leg with the tree trunk. However, the cracks arose more than three centuries after the statue had been unveiled (nineteenth century), hence the original David’s conception with its intrinsic weakness cannot be considered the main cause of the rising cracks. An elucidating explanation was recently offered by Borri and Grazini [2] who showed that the cracks in the legs were likely caused by a slight forward inclination of the statue arisen after the Florence flood of 1844 (another explanation is the additional weight placed on the David by Clemente Papi in 1847 to make a plaster cast). When in 1873 the David was transferred to the Accademia Gallery the tilting was corrected.

Even though the cracks do not seem to have worsened since the tilting correction, an experimental dynamic survey has been deemed opportune to evaluate the effect of the pedestrian-induced vibrations on the statue. The problem is relevant because, even if presently the cracks appear stable, the weakness of the David’s legs, as also demonstrated by some recent experimental results ([3]), can make the statue sensible to the damage induced by the walking of huge number of daily visitors. To assess this specific effect, during two days, Monday, 27^th^ and Tuesday, 28^th^ of July 2015, measurements were taken of the dynamic movements of Michelangelo's David by means of an interferometric radar. The first day (Monday) is closing day at the Accademia Gallery, while the second, a Tuesday, statistically is the peak attendance day.

Among the new technologies and methods employed for dynamic surveys ([4]), a microwave interferometer was used to assess the oscillations induced by ambient noise because a radar can: i) remotely detect movements without any mechanical contact with the observed object ([5–7]) and b) discriminate the behavior in different sections of the object in relation to their distance from the measuring instrument. The interferometric radar has been proved effective in measuring resonant frequencies by many monitoring surveys over the last years ([8]), mostly carried out on buildings (masonry towers [5–6, 9], bell-towers [10], wind towers, historic constructions [11]) or infrastructures (bridge [12], stay cables, railway culvert) where ambient loads (wind or traffic) are able to originate noticeable movements of the targeted structures. To the authors’ knowledge this is one of the first applications of the interferometric technique to detect movements of statues inside a building (i.e. where very small displacements are expected since ambient loads are almost absent).

During the investigation the radar was placed on a tripod that does not guarantee absolute immobility. So, to distinguish the possible movement of the instrument from the movement of the statue (thus calculating the effective displacement of the target), an accelerometer was installed on the radar head in the same direction of the antennas, which makes it an inertial station with respect to the measuring direction ([9, 13]). During the first measurement day (Gallery closing day), the movements of the statue were acquired from two different positions (front and side). This enabled to characterize the behavior of the structure along the two main symmetry directions. The next day, given the large number of visitors, it was possible to take the measurement only from the lateral position. The second measurement day was significant because it was used, by comparison with the previous one, to estimate the effect of visitors' attendance on the dynamic response of the statue.

## Materials and methods

To avoid any direct contact with the investigated object an interferometric radar was used in the experimental survey. The basic operating principle of radar sensor is to exploit the phase information of the electromagnetic waves echoed by the detected targets to obtain the local displacement. If the sampling time is short enough to follow the movements of the tested structure, its vibrations are detected as a phase rotation of the received radar signal. The instrument is equipped with two transmit/receive horn antennas, and operates at microwave frequency according to a technique referred to as continuous-wave step-frequency (CWSF) ([12]). Suitable processing of the acquired data provides a mono-dimensional range image of the scenario of interest: the motion of the target, producing a displacement along the radar-target axis, causes a proportional phase shift of the backscattered wave. The phase shift of the waves reflected before and after the target motion allows to obtain a mono-dimensional map of the displacements in the field of view.

The interferometric sensor is implemented as a portable equipment, installed on a tripod and powered by a battery pack. It radiates signals at 16.75 GHz center frequency with approximately 400 MHz bandwidth, thus providing 0.37-meter range resolution. The sensor is controlled via the USB port of a common notebook. Design specifications on signal accuracy and stability make the instrument suitable for measuring displacements with accuracy better than 0.1 mm. The use of high speed electronics enabled an acquisition rate up to 100 images per second.

In the following section, the results obtained in the two–day monitoring are discussed.

## Results and discussion

### Lateral position without visitors (first day)

**Fig 1:**
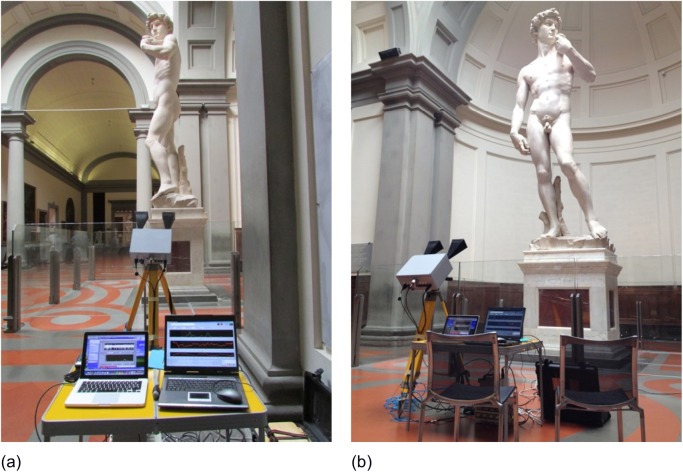
Measurement positions: (a) lateral; (b) frontal.

**Fig 2:**
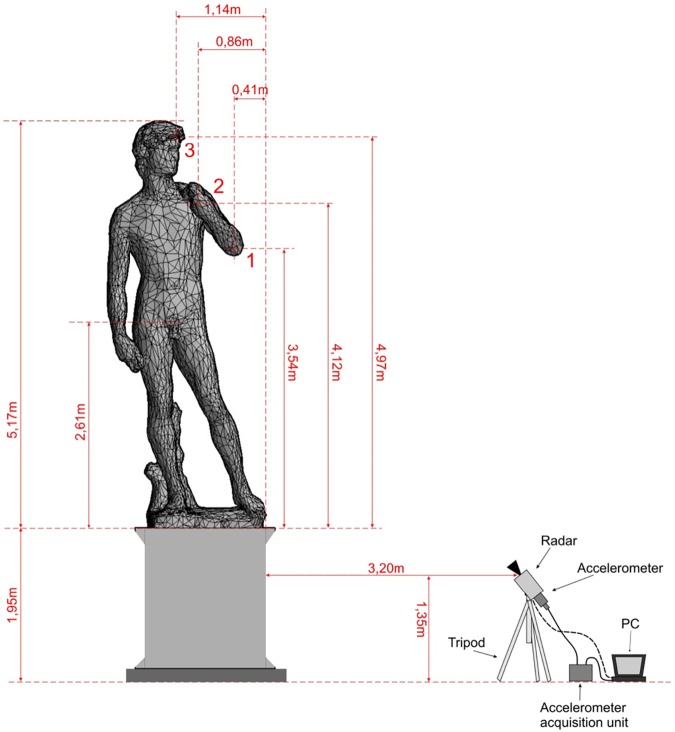
Measurement layout during lateral investigation. Zones {1, 2, 3} allowed to distinguish the interferometer echo.

As a first survey position, the radar was placed on the right side of the statue, about 3.2 m far from the pedestal, as shown in Fig 1A. In the radar image it was possible to distinguish the echo from the three areas, indicated with numbers 1, 2 and 3 in Fig 2. Zone 1 corresponds to the elbow of the left arm; zone 2 corresponds to the connection between the left hand of David and the sling; zone 3 corresponds to the David’s head.

**Fig 3:**
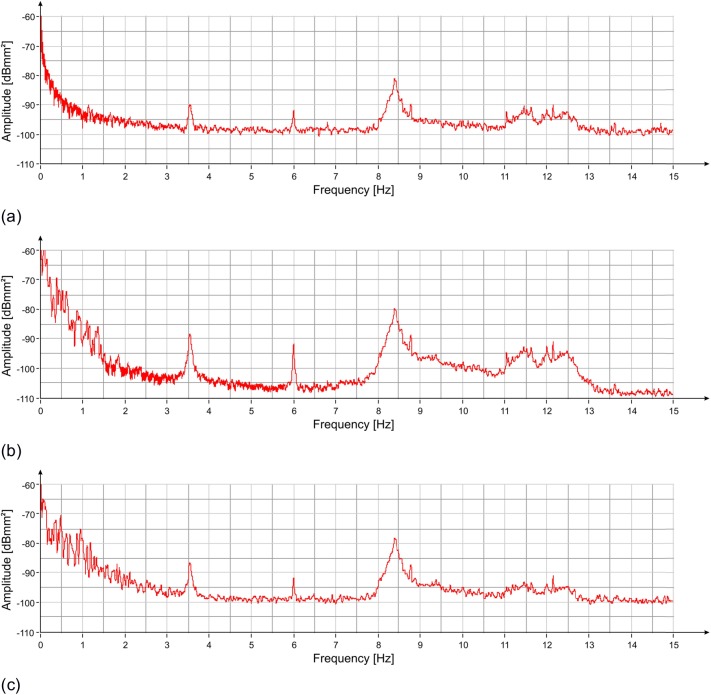
PS vs frequency of the displacement measured with the radar at three positions of the statue (Fig 2): (a) zone 1; (b) zone 2; (c) zone 3. Three resonant frequencies are visible at 3.55 Hz, 6.00 Hz and 8.4 Hz.

**Fig 4:**
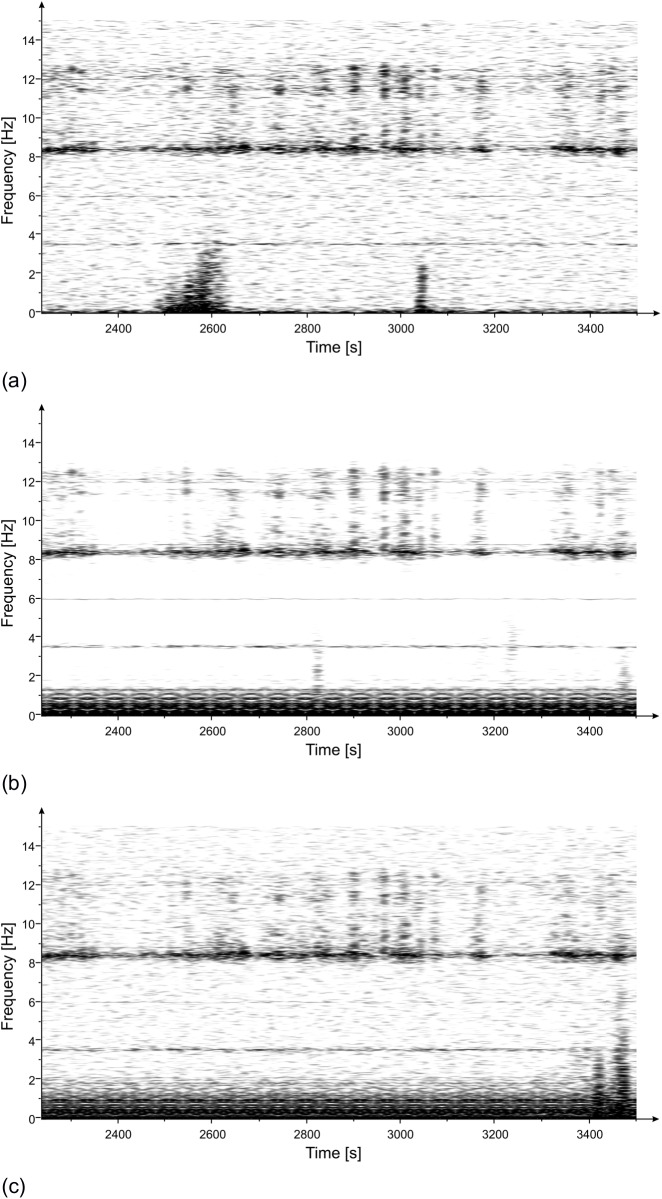
Frequency vs time of the displacement measured with the radar at three positions of the statue (Fig 2): (a) zone 1; (b) zone 2; (c) zone 3. Persistence over time of three frequencies (3.55 Hz, 6.00 Hz and 8.4 Hz) is visible.

The measurement lasted approximately 90 minutes. Acquired data were processed, and the Power Spectrum (PS) of the recorded signals (displacement) was evaluated using the modified Welch periodogram technique ([14]) by integrating over time-windows of 50 s, with 99% overlap between adjoining windows. The resulting PS, reported in Fig 3, clearly shows the presence of 3 distinct peaks at the following frequencies: 3.55 Hz, 6.00 Hz and 8.40 Hz. Also visible is a range of frequencies diffusively excited between 10.5 Hz and 12.5 Hz. The results of the spectral estimation on all the time-windows were joined to perform a Frequency versus Time analysis (JTFA), illustrated in Fig 4, that shows the persistence over time of the three above peaks.

**Fig 5:**
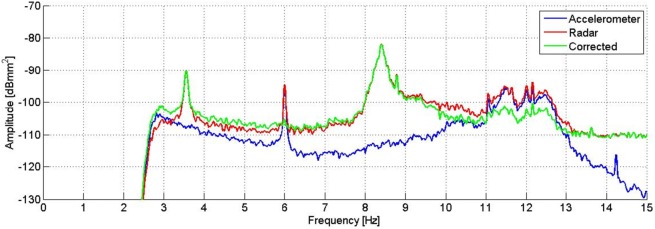
PS of the displacement measured by the radar (red) and the accelerometer (blue), and the result of the correction (green). The peak at 6 Hz disappears in the corrected signal, and the signals between 10.5 Hz and 12.5 Hz are attenuated.

As the instrument was installed on the ground, during the investigation it was stimulated like the statue. This means that, performing the instrument differential displacement measurement, the acquired data is the difference between the signal of the statue’s oscillations and the signal of the tripod’s oscillations in the line of sight between the radar and a scattering point. The measurement can hence be affected by the possible movement of the radar equipment. To filter out the tripod movements from the measurement, a seismic accelerometer (PCB Piezotronics, model 393B31) was fixed to the rear of the radar head during the survey. Its measurement axis was parallel to the radar line of sight, in order to detect only the component of the vibration that might affect the radar data. Sensitivity of the accelerometer is 1.02 V/m/s^2^, and its frequency range 0.1–200 Hz. It was connected to its acquisition unit by a coaxial cable. The displacement measured by the radar was converted to acceleration, and the accelerometer signal was subtracted in time domain according to a corrective procedure proposed by Pieraccini et al. [13]. After that, a PS analysis of the result of the displacement measured by the radar and corrected by the accelerometer was performed to identify the real resonance peaks of the statue, removing the resonance peaks due to the movement of the tripod. The correction technique allowed to eliminate the contribution of the resonance frequency at 6 Hz, and to attenuate the signal power of the frequencies between 10.5 Hz and 12.5 Hz. What remains after the filtering is the resonance frequency at 3.55 Hz and 8.40 Hz, as reported in Fig 5. It is also possible to observe a peak at 8.79 Hz which is not easily identifiable since, as reported next, it isn’t present in the measurements carried out the next day.

In order to better understand the origin of the resonance frequencies which are attenuated by the correction method (the peak at 6 Hz, and the signals in the range between 10.5 Hz and 12.5 Hz), an additional measurement was made by placing the accelerometer directly on the floor of the Accademia Gallery, near the radar station.

**Fig 6:**
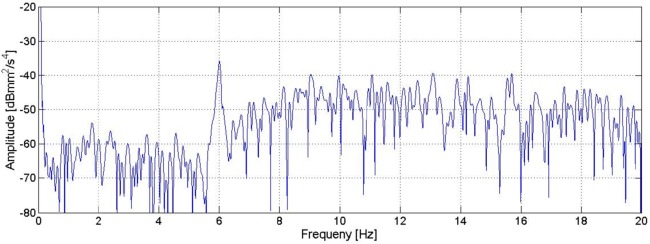
PS of the acceleration measured by the accelerometer placed directly on the floor of the Accademia Gallery, near the radar station.

Results of the signal analysis of this measurement are reported in Fig 6. The 6 Hz peak is clearly visible, while no significant contributions between 10.5 Hz and 12.5 Hz are observed. It is then possible to argue that the 6 Hz frequency (also measured by the radar), is due to artificial forcing present in the measuring environment (for example the HVAC at the Accademia Gallery), while the contribution of frequencies between 10.5 Hz and 12.5 Hz (visible only in the radar measurement) is due to the own motion of the tripod.

The length of each time-window employed to calculate the PS of the displacements (50 s) was selected to increase the frequency resolution, and to distinguish the different frequency peaks. However, the coherence of the oscillations was maintained for much shorter periods and the use of such an extended integration interval did not allow to correctly estimate the average power and, consequently, the average amplitude of the oscillations of the statue. If the integration period was reduced, the amplitude of the peaks increased, because the Fourier Transform was carried out in a time interval when the oscillation was more coherent. By reducing the integration interval recursively (i.e. the length of the time-window), it was possible to estimate when the width of the individual peaks tends to no further increase. In short, this time, represents the average coherence time of the oscillations to each resonance frequency; and the correspondent peak amplitude represents an estimation of the average amplitude of the oscillations of the statue. Doing so, the resulting peak at 3.55 Hz has a period of coherence of about 3 s, with power of -80 dBmm^2^. This corresponds to an amplitude of about 0.2 μm peak-to-peak. The frequency at 8.40 Hz has a period of coherence of about 1 s with power of -69 dBmm^2^, which corresponds to an amplitude peak-to-peak of about 0.7 μm.

**Fig 7:**
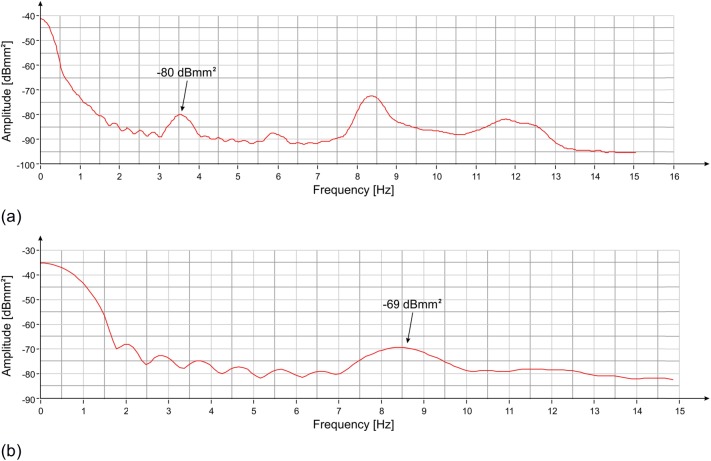
PS vs frequency of the displacement measured with the radar at position #3 (Fig 2): (a) 3 s time-windows (the amplitude of the corresponding 3.55 Hz peak is shown); (b) 1 s time-windows (the amplitude of the corresponding 8.40 Hz peak is shown).

**Fig 8:**
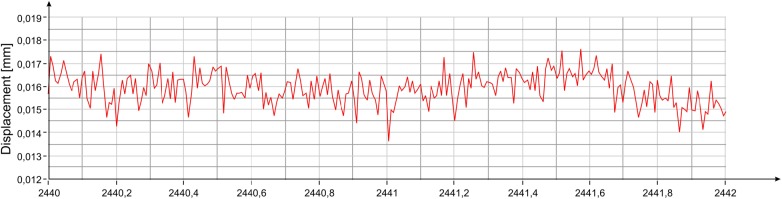
Displacement measured with the radar at position #3 (Fig 2).

The graphs of the spectral power with integration intervals of 3 s and 1 s are reported in Fig 7A and 7B, respectively. The amplitude of the oscillations estimated with the previous method approximately coincides with the peak-to-peak amplitude of the movement as measured by the radar (Fig 8). From simple mathematical operations it is also possible to estimate the average speeds of vibration along the direction of measurement corresponding to the two resonant frequencies of the statue, which are about 2 μm / s and about 18 μm / s, respectively.

The results obtained in the lateral position measurement in the absence of visitors are summarized in Table 1.

**Table 1:** Summary of the experimental results.

Lateral position (without visitors)		Frontal position (with visitors)		Lateral position (with visitors)	
Frequency [Hz]	Power [dBmm^2^]	Frequency [Hz]	Power [dBmm^2^]	Frequency [Hz]	Power [dBmm^2^]
3.55	-80	3.55	-72.5	3.54	-76
-	-	5.12	-	-	-
6.00	-	5.94	-	5.92	-
8.40	-69	8.40	-77	8.38	-69
8.79	-	8.79	-	-	-
10.5–12.5	-	11.05–11.88	-	10.5–12.5	-

### Frontal position without visitors (first day)

**Fig 9:**
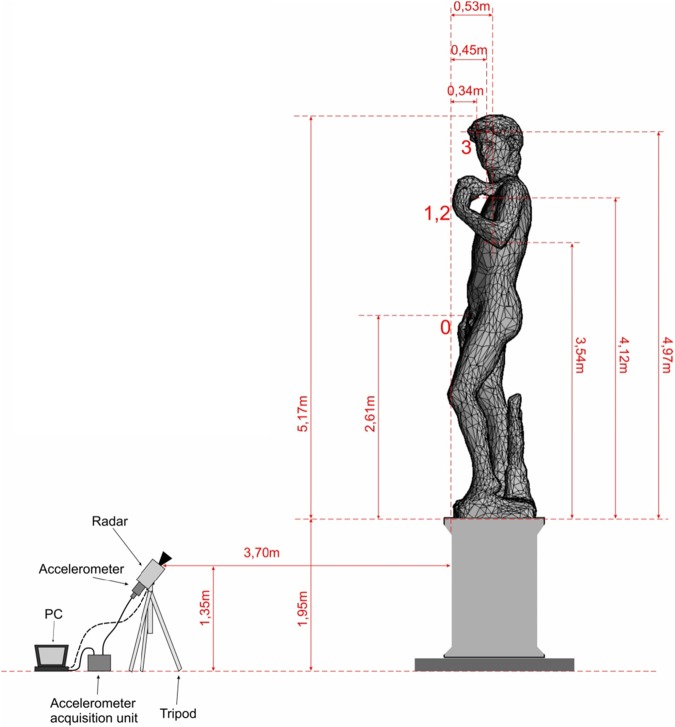
Measurement layout during frontal investigation. Zones {0, 1–2, 3} allowed to distinguish the interferometer echo.

In order to monitor the other symmetry direction of the statue, as a second survey configuration, the radar was positioned in front of the statue at about 3.7 m from the pedestal (Fig 1B). Measurements in this position were possible only on the first day, when the Accademia Gallery was closed. The radar image allowed distinguishing the echoes from three areas, indicated by the numbers 0, 1–2 and 3 in Fig 9. Zone 0 corresponds to the right hand and to the groin area; zone 1–2 corresponds to the left arm and the connection between the hand and the sling; zone 3 corresponds to David’s head.

**Fig 10:**
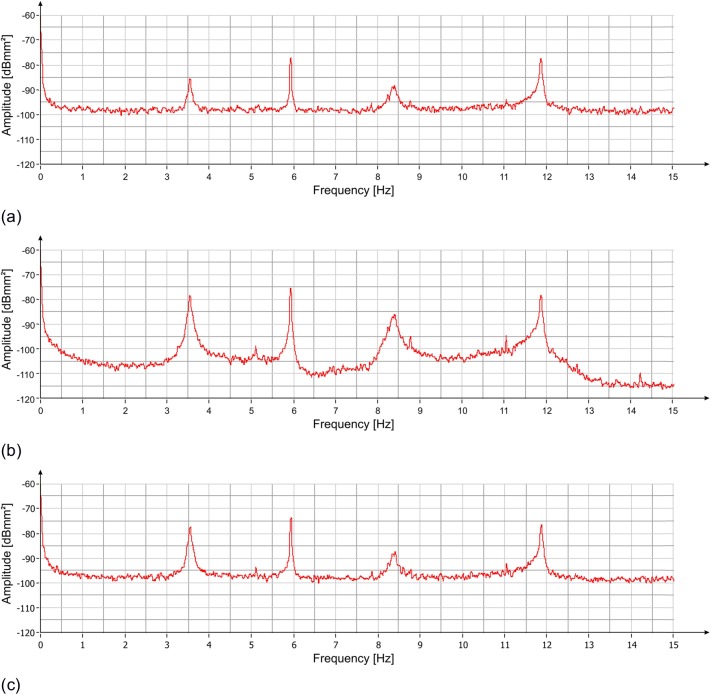
PS vs frequency of the displacement measured with the radar at three positions of the statue (Fig 9): (a) zone 0; (b) zone 1/2; (c) zone 3. Four resonant frequencies are visible at 3.55 Hz, 5.94 Hz, 8.40 Hz and 11.88 Hz.

**Fig 11:**
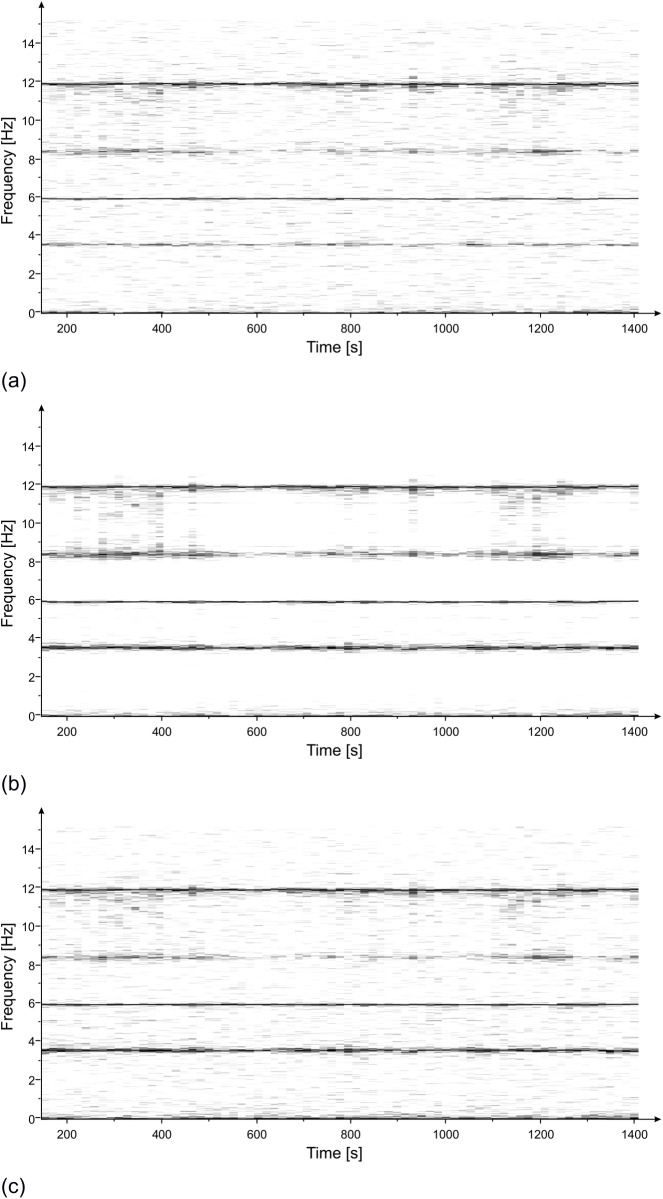
Frequency vs time of the displacement measured with the radar at three positions of the statue (Fig 9): (a) zone 0; (b) zone 1/2; (c) zone 3. Persistence over time of three frequencies (3.55 Hz, 5.94 Hz, 8.4 Hz and 11.88 Hz) is visible.

The PS of the measured displacements in the three positions, shown in Fig 9, was calculated by using the modified Welch periodogram technique and integrating over time-windows of 50 s with 66% overlap between adjoining windows. Results are illustrated in Fig 10 where four distinct peaks at the following frequencies: 3.55 Hz, 5.94 Hz, 8.40 Hz and 11.88 Hz are easily recognizable. The time-frequency analysis shows the persistence of these frequency peaks over the whole measurement time (Fig 11). In addition, the measurement of the displacement of the left arm area and of the connection between the hand and the sling, thanks to a better signal-to-noise ratio, allowed identifying other frequency contributions: 5.12 Hz, 8.79 Hz, 11.05 Hz and 14.24 Hz. The presence of the peak at 8.79 Hz was still recorded. This frequency, visible also during the lateral position measurement, remains an uncertain assessment due to its absence during the second measurement day.

**Fig 12:**
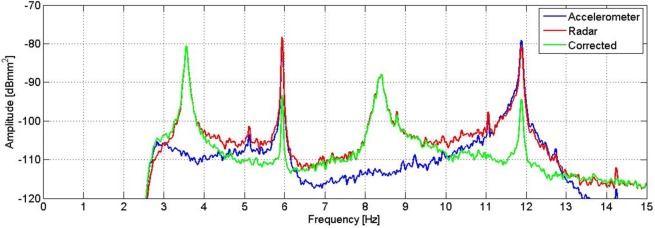
PS of the displacement measured by the radar (red) and the accelerometer (blue), and the result of the correction (green). The peaks at 5.94 Hz and 11.88 Hz are attenuated, while the ones at 5.12 Hz, 11.05 Hz and 14.24 Hz disappear after the correction of the signal.

The correction of the radar measurements by means of the accelerometric signal highlights the effective natural frequencies of the statue and those arising from the vibrations of the measuring instrument (tripod). From the estimation of the spectral power (Fig 12), it is possible to obtain the actual frequencies of the statue: the ones at 3.55 Hz and 8.40 Hz. On the green curve of Fig 12, it is possible to observe that the peak amplitudes at 5.94 Hz and 11.88 Hz, after the radar measurement correction by means of the accelerometric signal, are attenuated by a factor of about 15 dB. It is interesting to note the permanence of few residual peaks; this is essentially due to the limitations of the employed correction technique. One of the causes is the lack of amplitude constancy of the frequency response of the accelerometer, which causes the uneven amplification of the different frequency contributions. Generally speaking, the adopted correction technique by means of accelerometric measurements allows good results when the measured object and the measuring instrument exhibit completely independent movements. If the frequency of 6 Hz, certainly due to artificial forcing, was able to cause movements albeit small both on the radar and on the statue, these movements cannot be corrected in an optimal way by the employed technique. For the reasons listed above, it is therefore reasonable to assume that both frequencies that are attenuated (but not eliminated) by the correction technique may not represent natural vibration modes of the statue, although there are still residual peaks after correction.

**Fig 13:**
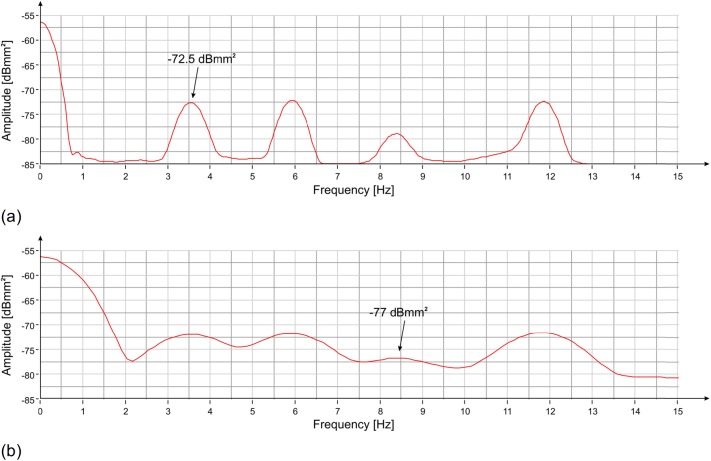
PS vs frequency of the displacement measured with the radar at position #3 (Fig 9): (a) 3 s time-windows (the amplitude of the corresponding 3.55 Hz peak is shown); (b) 1 s time-windows (the amplitude of the corresponding 8.40 Hz peak is shown).

**Fig 14:**
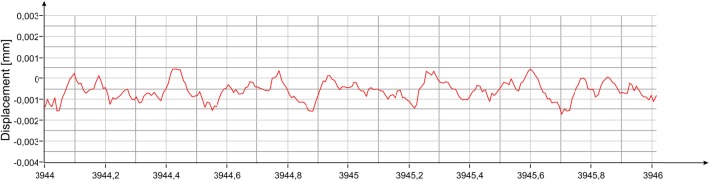
Displacement measured with the radar at position #3 (Fig 9).

As has been done for the measurement from the lateral position, the estimation of the oscillation power of the two main frequency contributions of the statue was obtained by reducing the length of the integration intervals to bring them closer to the correspondent coherence times. Fig 13A shows how the oscillation power of the 3.55 Hz frequency is approximately -72.5 dBmm^2^, corresponding to about 0.5 μm peak-to-peak. Fig 13B shows that at the frequency of 8.40 Hz, the oscillation power is between -78 dBmm^2^ and -77 dBmm^2^, corresponding to about 0.3 μm peak-to-peak. The uncertainty is due to the fact that the decrease in integration time results in a considerable reduction of the resolution and in this second case the amplitude of the peak begins to undergo the influence of the tails of the neighboring peaks. The displacement measured by the radar for a two-second interval is shown in Fig 14.

Also in this case it is possible to estimate the average speed of vibration in the measurement direction for the two frequency contributions, which corresponds to about 6 μm / s and 8 μm / s, respectively.

Table 1 summarizes results of the frontal position measurement with no audience.

### Lateral position with visitors (second day)

During the second day (opening day), for safety reasons, the measurement was performed from the lateral position only. The interferometric radar was positioned in the same measurement location as the previous day (Fig 1A). Again the radar was able to detect the echo of the three areas indicated with numbers 1, 2 and 3 in Fig 2.

**Fig 15:**
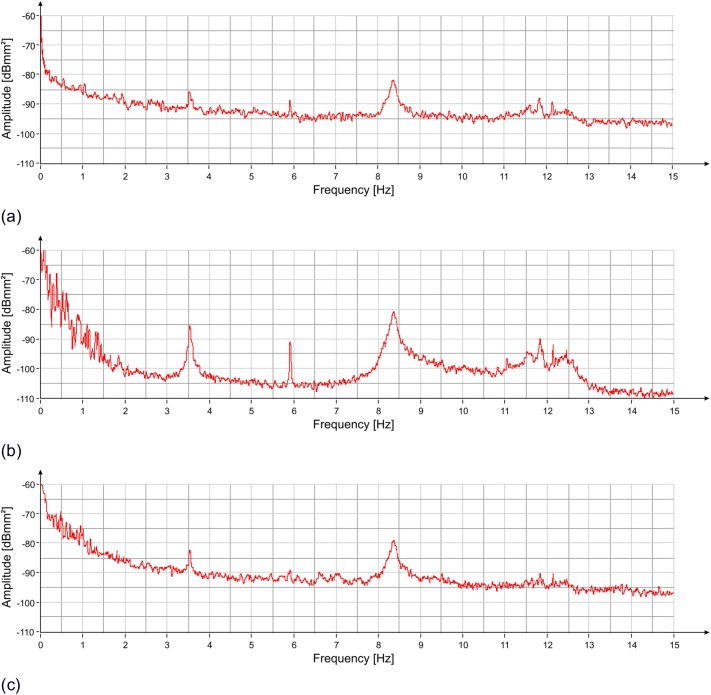
PS vs frequency of the displacement measured with the radar at three positions of the statue (Fig 2): (a) zone 1; (b) zone 2; (c) zone 3. Three resonant frequencies are visible at 3.54 Hz, 5.92 Hz and 8.38 Hz.

**Fig 16:**
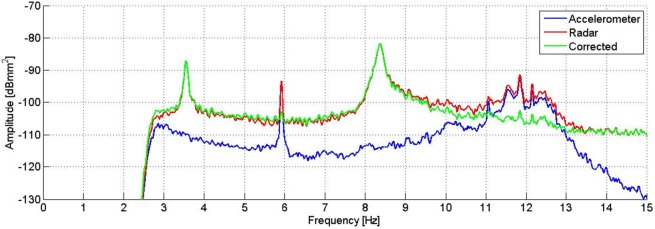
PS of the displacement measured by the radar (red) and the accelerometer (blue), and the result of the correction (green). The peak at 6.0 Hz disappears, and the signals between 10.5 Hz and 12.5 Hz are attenuated after the correction of the signal.

The estimation of the PS, evaluated using the modified Welch periodogram technique by integrating over time-windows of 50 s with 99% overlap between adjoining windows, is shown in Fig 15. The spectral analysis of the measured displacements shows a result similar to the one obtained on the previous day. The only remarkable difference is the absence, in this case, of the peak at 8.79 Hz. The measured resonance frequencies are: 3.54 Hz, 5.92 Hz and at 8.38 Hz. After applying the correction procedure, the peak at 5.92 Hz disappears, and the frequency contribution between 10.5 and 12.5 Hz is considerably attenuated (Fig 16).

**Fig 17:**
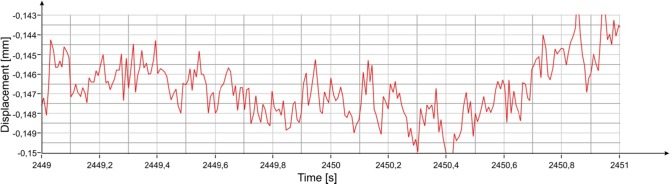
Displacement measured with the radar at position #3 (Fig 2).

The comparison between peak amplitudes reported in Fig 16 and Fig 5, shows that in the presence of the audience, the amplitude of oscillations at the frequency of 3.54 Hz has increased of about 4 dB while that at 8.38 Hz remains substantially unchanged. It is worth mentioning that the dB is a logarithmic scale and an increase of 4 dB corresponds to an increase in amplitude in the linear scale of about 60%. The estimated average power for the frequency 3.54 Hz, steps from -80 dBmm^2^ to -76 dBmm^2^, corresponding to an amplitude of about 0.3 μm peak-to-peak. On the contrary, the average power for the 8.38 Hz frequency, will remain unchanged at the value of -69 dBmm^2^, corresponding to 0.7 μm peak-to-peak. The displacement measured by the radar for a 2 s interval is reported in Fig 17. It is possible to estimate the average speed of vibration in the measuring direction for the two frequency contributions, which corresponds to about 3 μm / s and 18 μm / s, respectively.

The results obtained in the lateral position measurement with audience are summarized in Table 1.

### Numerical modelling

The finite element (FE) software CODE ASTER was employed to build the numerical model of Michelangelo’s David. The geometry model was created according to the laser scanner survey performed within the “Digital Michelangelo Project” coordinated by Marc Levoy (Stanford University) and developed between 1997 and 1999. FE model was built with 147,323 isoparametric three-dimensional (3D) elements and 36,728 nodes. The CODE ASTER model was employed to perform linear modal analyses, compare the experimental results and investigate the reliability of the frequency at 8.79 Hz detected the during the first measurement day.

**Fig 18:**
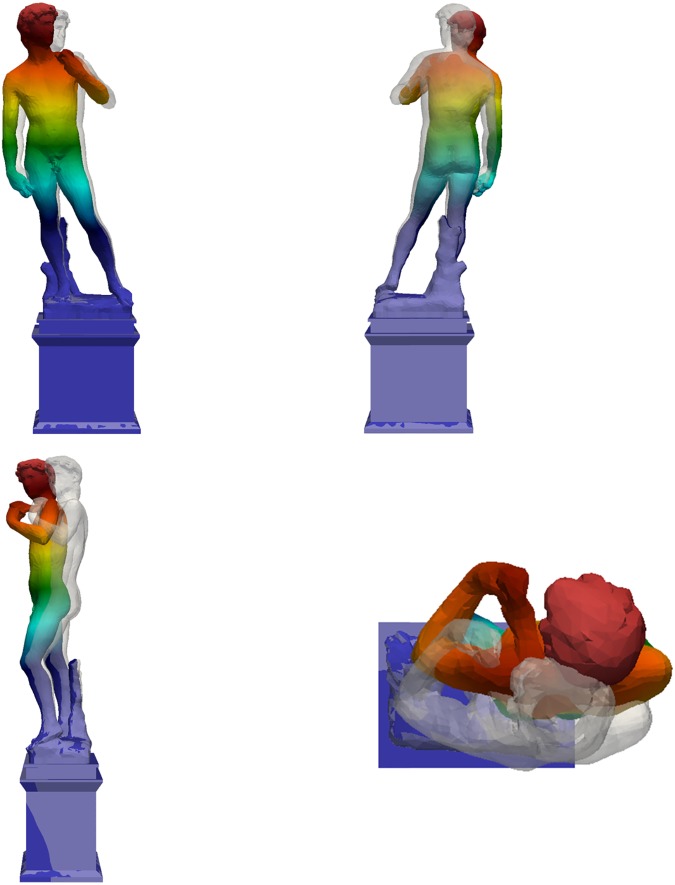
First mode shape of the David (different colors refer to different displacement amplitudes): f_1,Num_ = 3.55 Hz.

**Fig 19:**
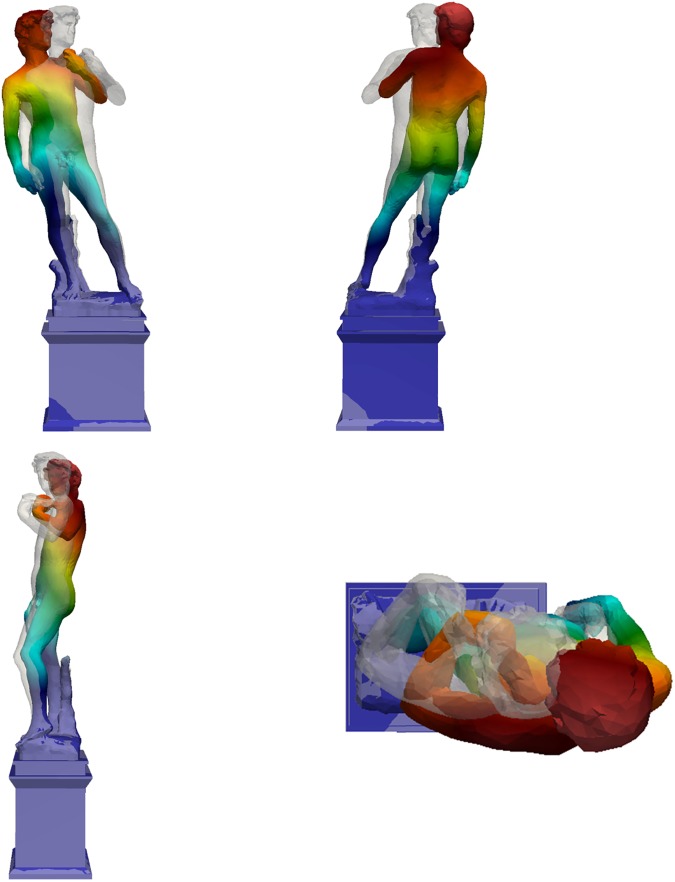
Second mode shape of the David (different colors refer to different displacement amplitudes): f_2,Num_ = 8.39 Hz.

**Fig 20:**
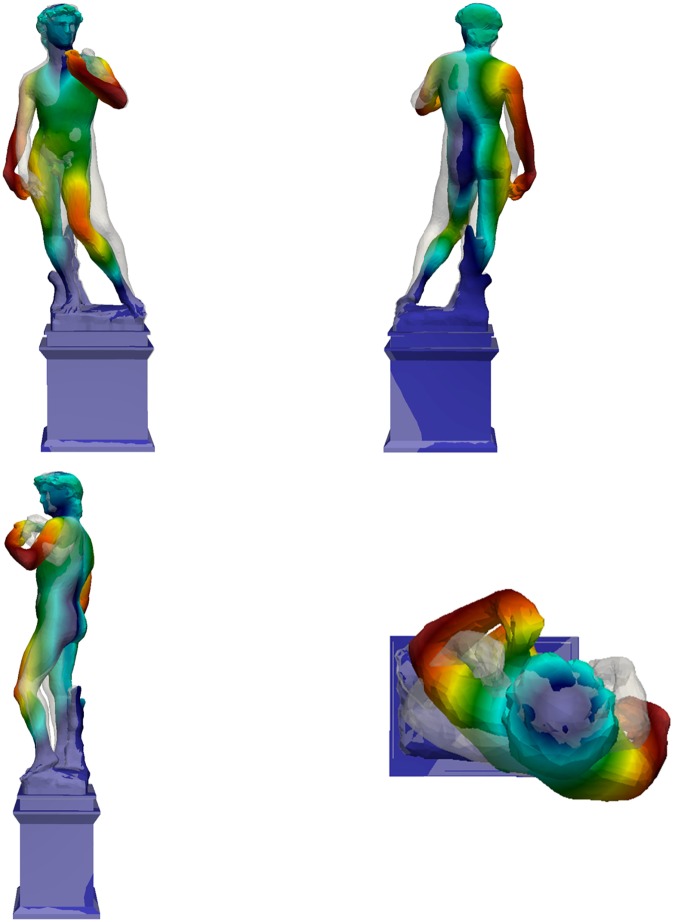
Third mode shape of the David (different colors refer to different displacement amplitudes): f_3,Num_ = 23.11 Hz.

Mechanical parameters of the numerical model (mainly the elastic modulus and the own weight) were parametrically tuned to compare the results of dynamic simulation with those of the experimental campaign based on commonly employed back-identification procedures [15]. In particular, to assess the dynamic effects, the elastic modulus of the marble materials was varied within its admissible range, together with the elastic modulus of the pedestal, until the first two experimental frequencies were reproduced. The first three numerical mode shapes of Michelangelo’s David are reported in Fig 18–Fig 20. The first mode shape (Fig 18) is a bending mode along the weak direction of the statue with a frequency of 3.55 Hz, the second mode (Fig 19) is still a bending one, in the lateral direction, with a frequency of 8.39 Hz. The third mode (Fig 20) is a torsional one, with a frequency value of 23.11 Hz. Higher modes, not reported here, are local modes that activate partial movements of the statue (the leg, the arm, the head, etc.). The numerical results identify the experimental frequencies and allow to exclude the experimental value of 8.79 Hz (obtained only the first measurement day) as a frequency of interest of the statue.

## Conclusions

This paper reports a study on Michelangelo’s David based on radar measurements and data analysis that allowed to identify the dynamic behavior of the statue by only using ambient noise. To evaluate the effects of the pedestrian loading induced by visitors, the radar measurements were taken on a closing day of the museum, and repeated on the second opening day (statistically the day with higher tourist affluence). Given the measurement uncertainty, the frequencies with values between 3.54 Hz and 3.55 Hz were associated with the same mode of vibration of the statue with a univocal value of 3.55 Hz. Frequencies with values between 8:38 Hz and 8.40 Hz were associated with another mode of vibration, and the value was attributed to 8.40 Hz. The experimental frequencies were confirmed by a numerical model of the statue, and by FE model one frequency value (obtained only on the first measurement day was excluded as a frequency of interest of David. After tuning, the FE model is a good candidate for future investigations (to assess the statue structural behavior under severe loading such as seismic loading). However, further targeted experimental investigations are required before (f.i. to evaluate the pedestal structural details).

With respect to the effects of the pedestrian-induced vibrations on the dynamic response of Michelangelo's David, the average recorded amplitudes of the statue movement on the closing day, evaluated at the resonance frequencies, were found not to exceed 1 μm peak-to-peak (corresponding to 0.001 mm peak-to-peak) while the average vibration speed did not exceed 20 μm / s (corresponding to 0.02 mm / s). The presence of the audience has affected only the lower resonance frequency, thus increasing the average amplitude of the displacement of about 60%. Despite this increase, the absolute value of the average displacement amplitude remains small. The amplitude of the statue oscillations due to forcing machinery (e.g. the HVAC), if any, was not possible to estimate since artificial forcing causes the movement of both the measuring instrument and the target.
